# Assessing eco-environmental quality and its drivers in the Shandong section of the Yellow River Basin with an improved remote sensing ecological index

**DOI:** 10.1038/s41598-025-31580-3

**Published:** 2025-12-28

**Authors:** Peipei Wang, Chun-Pin Tseng, Yiyou Fan, Qinghao Wei, Min Qiao, Xiaoshuang Li, Ran An

**Affiliations:** 1 Civil Engineering Department, Qilu Institute of Technology, Jinan, 250200 China; 2 Mathematics Department, Brandeis University, Waltham, MA USA

**Keywords:** The Shandong section of the Yellow River Basin (SDYRB), Eco-environmental quality, Improved remote sensing ecological index (IRSEI), Spatial autocorrelation, Driving factors, Ecology, Ecology, Environmental sciences

## Abstract

The Shandong section of the Yellow River Basin (SDYRB), a critical zone for ecological security in the lower reaches of the Yellow River, faces multiple ecological challenges including salinization, soil erosion, water scarcity, and anthropogenic pollution. These issues significantly hinder regional sustainable development. To assess eco-environmental quality in the SDYRB accurately, an Improved Remote Sensing Ecological Index (IRSEI) was developed by integrating the Composite Salinity Index (CSI) and Soil–Water Conservation Function Index (SWCFI). Utilizing multi-temporal imagery (2009–2023), this study analyzed spatio-temporal patterns of eco-environmental quality and their driving mechanisms. The results show that: (1) The overall eco-environmental quality exhibits a declining trend, with a spatial distribution pattern characterized as “superior in the west and poorer in the east”. High-quality areas were concentrated in western plains and Yellow River riparian zones, versus low-quality areas in eastern/northern coasts. (2) The global Moran’s I approached 1 and exhibited a gradual year-by-year decline, indicating persistent spatial agglomeration of ecological quality. Local spatial autocorrelation was predominantly characterized by High-High (H–H) and Low-Low (L–L) agglomerations, with low-value areas exhibiting an outward spread tendency. (3) Ecological quality fluctuated, declining significantly (2009–2014) before recovering (2019–2023). Degradation hotspots were identified in the northeast and southwest, whereas the improved areas were concentrated in the central region. (4) Ordinary Least Squares (OLS) regression and GeoDetector (GD) identified synergistic natural and anthropogenic driving factors: mean annual temperature, evapotranspiration, nighttime light intensity, and land use were dominant. This study improves the applicability and interpretability of IRSEI in salinized and soil-eroded regions by integrating CSI and SWCFI, offering a scientific foundation for ecological conservation and high-quality development in the SDYRB. The approach can also be extended to dynamic monitoring and evaluation of other similarly vulnerable ecological zones.

## Introduction

As the mother river of China, the Yellow River Basin is of profound strategic importance for the country’s economic and social development and ecological security^1^^.^ The Shandong section constitutes a vital area within the lower reaches of the Yellow River. It not only possesses rich natural resources and a unique geographical environment but also plays a pivotal role in maintaining ecological balance and ensuring regional ecological security^2^. However, alongside rapid regional economic development, the eco-environment of SDYRB faces numerous challenges^3^, including water scarcity^4^, soil erosion^5^, and industrial and agricultural pollution^6^. In particular, the widespread problems of soil salinization and ecological fragility in its coastal zones seriously threaten the ecological health of the basin.

To systematically address these challenges, the country attaches great importance to the sustainable development and ecological governance of the Yellow River Basin. In September 2019, A symposium on ecological protection and high-quality development in the Yellow River Basin was held in Zhengzhou. During this symposium, the major national strategy of ecological protection and high-quality development in the Yellow River Basin was formally proposed^7^. In 2021, the CPC Central Committee and the State Council officially issued the Outline of the Plan for Ecological Protection and High-quality Development in the Yellow River Basin. It further emphasized that modern scientific and technological means should be employed to strengthen ecological protection and environmental governance and to promote the green transformation of the basin^8^. As the only province among the nine provinces and autonomous regions along the Yellow River that has both the geographical features of being along the Yellow River and being on the coast^9^, Shandong occupies a special and important position in this strategy. Therefore, it is essential to conduct a precise assessment of the ecological environment quality in the SDYRB. This is critical for ecological risk early warning, the development of protective measures, and the optimization of regional development planning^10^.

Currently, the assessment of ecological environment quality predominantly relies on ground-based monitoring data, which suffers from limitations such as restricted spatiotemporal coverage and high costs^11,12^. Remote sensing technology, with its advantages of broad spatial coverage, high efficiency, and continuous dynamic monitoring capability, has been extensively used in ecological assessment practices^13,14^. Current eco-environmental monitoring utilizing remote sensing data primarily employs the following approaches. Key remote sensing indicators are utilized, including vegetation indices^15^ (e.g., the Normalized Difference Vegetation Index (NDVI) and the Enhanced Vegetation Index (EVI)), the Normalized Difference Bare Soil Index (NDBSI), the Modified Normalized Difference Water Index (MNDWI)^16^, and Land Surface Temperature (LST). Meanwhile, integrating environmental driving factors (e.g., precipitation and other climatic variables), enables dynamic monitoring and analysis of the regional eco-environment. However, a single indicator is inadequate to fully capture the overall status of the regional eco-environment. Consequently, comprehensive models such as the Remote Sensing Ecological Index (RSEI)^17^ have gradually emerged as mainstream evaluation tools and have been effectively applied in various regions. For instance, Yan et al.^18^ elucidated ecological evolution patterns in Jiawang District. Similarly, Li et al.^19^ identified that the improvement of ecological quality in the Shendong Mining Area was associated with vegetation restoration. Zhang et al.^20^ quantified eco-environmental quality improvements in Gulang County through integrated RSEI and stepwise regression analysis. Li et al.^21^ observed decelerated degradation rates in Wuhan from 2005 to 2015, identifying spatial clustering of degraded zones in urban cores. Yao et al.^22^ developed an RSEI model based on Google Earth Engine (GEE), demonstrating upward ecological trajectories in the Hotan Oasis and confirming its applicability in arid regions.

Nevertheless, the traditional RSEI has notable shortcomings when applied to the SDYRB. On one hand, approximately 70% of the coastal wetlands in this region are affected by salinization stress^23^, while traditional RSEI indicators such as greenness and wetness fail to adequately capture the damage inflicted by salinity on the ecological environment. On the other hand, soil erosion poses a serious challenge in the piedmont alluvial plains and the estuarine delta, yet the conventional model does not systematically incorporate soil and water conservation functions^24^. In terms of driving force analysis, most existing studies focus on the isolated effects of individual factors^25,26^, while overlooking the interactions between natural and anthropogenic factors, which limits the depth of mechanism analysis. Thus, how to effectively integrate remote sensing data and ecological indices for a accurate assessment of ecological quality in the SDYRB remains a challenge worthy of further in-depth investigation.

To accurately evaluate the ecological quality of the SDYRB and uncover its spatiotemporal evolution patterns and driving mechanisms, this study will address the following three core questions: (1) How can an improved remote sensing ecological index (IRSEI) be constructed that effectively responds to both salinization stress and soil erosion, thereby enhancing its applicability in coastal vulnerable ecological regions? (2) What are the spatiotemporal evolution characteristics of the ecological environmental quality in this region between 2009 and 2023? (3) How do natural and anthropogenic factors drive changes in ecological environmental quality through both independent and interactive effects? Based on these questions, this study sets forth three research objectives: (1) By integrating the Composite Salinity Index (CSI) and theSoil-Water Conservation Function Index (SWCFI), the traditional RSEI model was enhanced to develop an IRSEI index tailored for the SDYRB. (2) Using multi-temporal remote sensing images, methods such as spatial autocorrelation and transition matrix were used to systematically uncover the spatiotemporal variations and evolution patterns of ecological quality in the SDYRB from 2009 to 2023. (3) By integrating multidimensional natural and human-driven factors, Ordinary Least Squares (OLS) and GeoDetector (GD) were applied to quantitatively analyze the independent impacts and interactive effects of each factor, so as to reveal the underlying driving mechanisms. This study aims to provide a scientific basis for ecological management and high-quality development in the SDYRB, and to offer a transferable methodological reference for the dynamic monitoring and assessment of similar ecologically vulnerable regions.

## Study area and data sources

### Study area

**Fig. 1:**
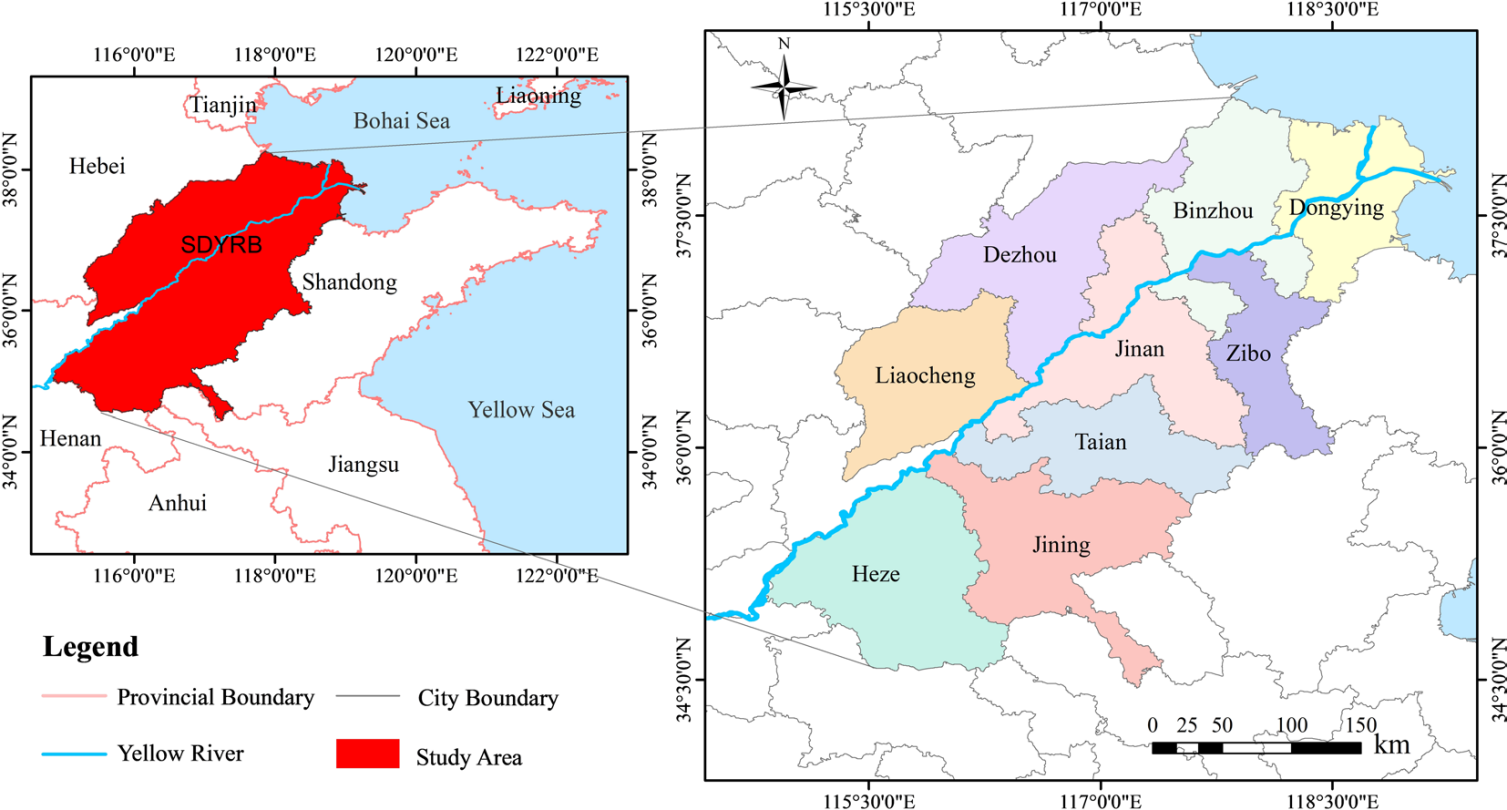
Location of the study area.

SDYRB (Fig. 1) constitutes a strategically significant region in the lower reaches, characterized by distinctive geographical attributes and abundant natural resources. Extending westward from Dongming County, the SDYRB traverses nine cities—Heze, Jining, Tai’an, Liaocheng, Jinan, Dezhou, Binzhou, Zibo, and Dongying—ultimately empties into the Bohai Sea at Kenli District, Dongying City. This section spans approximately 628 km, accounting for 11.5% of the Yellow River’s total length.

SDYRB represents the exclusive confluence zone where the Yellow River meets the sea, endowing it with critical ecological functions and strategic significance^27^. Notably, it sustains the Yellow River Delta’s most intact warm-temperate wetland ecosystem, playing a vital role in safeguarding ecological security throughout the lower Yellow River and adjacent Bohai Sea^28,29^. Regional wetlands encompass 1.2 million hectares, constituting 70% of Shandong’s total wetland area. Forest coverage extends over 1.13 million hectares, accounting for 40% of the province’s total. Additionally, the region hosts multiple national and provincial nature reserves.

However, SDYRB faces significant challenges, including demanding ecological restoration tasks, substantial flood-control pressures, and acute water resource constraints^30^. In response to these challenges, Shandong Province has formulated a strategic plan for ecological protection and high-quality development, aiming to strengthen ecosystem management, ensure river stability, promote sustainable basin-wide development, and enhance human well-being^31^.

### Data sources

NDVI, LST, and SWCFI were derived from MOD13A1, MOD11A2, and the Earth Resources Data Cloud, respectively. WET, NDBSI, and CSI were primarily calculated using Landsat imagery obtained from the United States Geological Survey (USGS) official platform. Summer datasets (June to August) for 2009, 2014, 2019, and 2023 were acquired, maintaining consistent temporal intervals and cloud cover of less than 5%. Specifically, 2009 data originated from the Landsat 5 Thematic Mapper (TM), whereas 2014–2023 data were obtained from the Operational Land Imager (OLI) sensor aboard Landsat 8. All acquired images correspond to Collection 2 Level-2 (C2 L2) products. Detailed information is documented in Table 1.

**Table 1:** Introduction of remote sensing datasets.

Data type	Year	Path row number	Resolution(m)	Data source
TM	2009	121/34,121/35,122/34,122/35, 122/36,123/34,123/35,123/36	30	The United States geological survey (USGS) (https://earthexplorer.usgs.gov/)
OLI/TIRS	2014, 2019, 2023			
MOD13A1	2009, 2014, 2019, 2023	h27v05	500	Google earth engine (GEE) (https://code.earthengine.google.com/)
MOD11A2		h27v05	1000	
SWCFI		–	1000	Earth resources data cloud (ERDC) (www.gis5g.com)

Regional eco-environmental quality arises from synergistic interactions between natural and anthropogenic elements. Drawing on prior studies^32–34^ and the regional attributes of the SDYRB, nine indicators were selected, including mean annual temperature, mean annual evapotranspiration, land use type, and nighttime light intensity, and five additional indicators (detailed in Table 2), thus establishing an eco-environmental quality driving factor system. Data sources for each variable are cataloged in Table 2. To address differences in data types, sources, and spatial resolutions^35,36^, all nine indicators were uniformly processed through clipping and resampling to generate 1-km resolution raster datasets.

**Table 2:** Data acquisition of IRSEI and its influencing factors.

Type		Variable	Year	Resolution (m)	Data availability
Dependent variable (Y)		IRSEI	2009, 2014, 2019, 2023	1000	Calculated from a combination of six indicators: NDVI, WET, LST, NDBSI, CSI, SWCFI
Independent variable (X)	Natural factors	Temperature	2023	1000	National Earth System Science Data Center (https://www.geodata.cn/main/)
		Evapotranspiration	2023	1000	
		Precipitation	2023	1000	
		Slope	2019	30	Obtain DEM data based on ASTER GDEM V3 dataset, extract slope based on DEM data
		Soil Moisture	2022	1000	National Tibetan Plateau / Third Pole Environment Data Center (https://data.tpdc.ac.cn/)
	Anthropogenic factors	Land use type	2023	30	Wuhan University CLCD land cover dataset
		Population Density	2023	1000	LandScan 2023 Global Population Dataset (https://landscan.ornl.gov/)
		Density of scenic spots	2023	1000	Scenic spots are obtained from the POI data of AutoNavi Maps POI data and calculated by kernel density analysis to get the results
		NPP-VIIRS-like nighttime light data	2023	500	National Earth System Science Data Center (https://www.geodata.cn/main/)

## Methods

### Research route

**Fig. 2:**
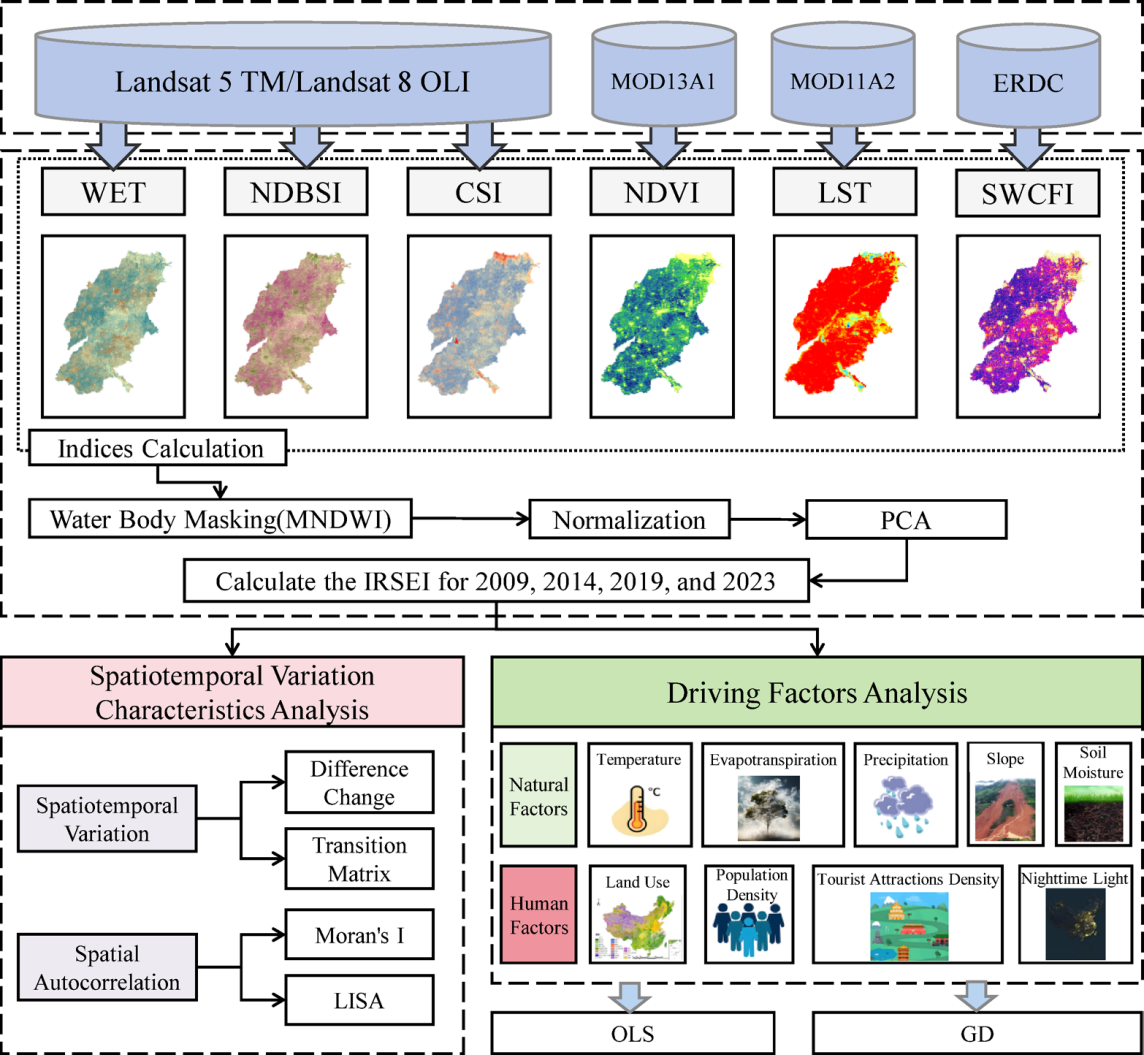
Framework of this study.

The framework is presented in Fig. 2. First, Landsat satellite images for four time periods (2009, 2014, 2019, and 2023) were acquired from USGS to serve as data sources for extracting humidity, dryness, and salinity indicators within the SDYRB. Subsequently, greenness, heat, and SWCFI, acquired from GEE and ERDC, were integrated. These six indicators underwent water body masking and normalization. Subsequently, the IRSEI model was constructed using PCA. The spatio-temporal evolution characteristics of IRSEI across the different time periods were then analyzed using the following methods: difference change analysis, transfer matrix, global Moran’s I, and LISA cluster maps. Finally, leveraging the OLS and GD models, the key driving factors were identified, and the spatial heterogeneity associated with these factors was explored.

### Construction of the IRSEI index

1. Remote sensing index calculation

Based on the IRSEI proposed in the previous text, six indices, namely NDVI, WET, NDBSI, LST, CSI, and SWCFI, were calculated. Referring to relevant studies^37,38^, calculation methods for WET, NDBSI, and CSI are shown in Table 3. Formula 1 is the expression of IRSEI:

10 $$IRSEI = f\left( {NDVI, WET, LST, NDBSI, CSI, SWCFI} \right)$$

where ${\mathrm{IRSEI}}$ is the Improved Remote Sensing Ecological Index; ${\mathrm{NDVI}}$ is the greenness index; ${\mathrm{WET}}$ is the humidity component; ${\mathrm{LST}}$ is the heat index; ${\mathrm{NDBSI}}$ is the dryness index; ${\mathrm{CSI}}$ is the salinity index; and ${\mathrm{SWCFI}}$ is the soil and water conservation function index.

**Table 3:** Indicators and calculation methods.

Index	Calculation formula	Number	Parameter meaning
WET	$WET_{TM} = 0.0315\rho_{blue} + 0.2021\rho_{green} + 0.3102\rho_{red} + 0.1594\rho_{nir} - 0.6806\rho_{swir1} - 0.6109\rho_{swir2}$	(1)	$\rho_{nir}$, $\rho_{red}$, $\rho_{blue}$, $\rho_{green}$, $\rho_{swir1}$, $\rho_{swir2}$ are the reflectance of near-infrared band, red band, blue band, green band, short-wave infrared 1, and short-wave infrared 2 bands, respectively ${\mathrm{SI}}$ is the bare soil index; ${\mathrm{IBI}}$ is the applied building index $\mathrm{SI-T}$, ${\mathrm{SI3}}$ and ${\mathrm{NDSI}}$ is the salinity index
	$$WET_{OLI} = 0.1511\rho_{blue} + 0.1973\rho_{green} + 0.3283\rho_{red} + 0.3407\rho_{nir} - 0.7117\rho_{swir1} - 0.4559\rho_{swir2}$$	(2)	
NDBSI	$\mathrm{NDBSI} = \frac{\mathrm{SI+IBI}}{2}$	(3)	
	$$SI = \frac{{\left( {\rho_{swir1} + \rho_{red} } \right) - \left( {\rho_{nir} + \rho_{blue} } \right)}}{{\left( {\rho_{swir1} + \rho_{red} } \right) + \left( {\rho_{nir} + \rho_{blue} } \right)}}$$	(4)	
	$$IBI = \frac{{\frac{{2\rho_{swir1} }}{{\rho_{swir1} + \rho_{nir} }} - \left( {\frac{{\rho_{nir} }}{{\rho_{nir} + \rho_{red} }} + \frac{{\rho_{green} }}{{\rho_{green} + \rho_{swir1} }}} \right)}}{{\frac{{2\rho_{swir1} }}{{\rho_{swir1} + \rho_{nir} }} + \left( {\frac{{\rho_{nir} }}{{\rho_{nir} + \rho_{red} }} + \frac{{\rho_{green} }}{{\rho_{green} + \rho_{swir1} }}} \right)}}$$	(5)	
CSI	$\mathrm{CSI} = \frac{\mathrm{SI-T+NDSI+SI3}}{3}$	(6)	
	$$SI - T = (\rho_{red} - \rho_{nir} \} ) \times 100$$	(7)	
	$$NDSI = \frac{{\rho_{red} - \rho_{nir} }}{{\rho_{red} + \rho_{nir} }}$$	(8)	
	$$SI3 = Sqrt\left( {\rho_{green}^{2} + \rho_{red}^{2} } \right)$$	(9)

(2) Water body identification and masking

The MNDWI^16^ was applied for water body masking. The calculation formula is as follows.

11 $$MNDWI = \frac{{\rho_{green} - \rho_{swir1} }}{{\rho_{green} + \rho_{swir2} }}$$

In the formula, $\rho_{green}$ and $\rho_{swir1}$ represent the green band and the shortwave infrared 1 band corresponding to the Landsat image, respectively.

(3) Normalization

Given dimensional inconsistencies and magnitude variations among the six indices, min–max normalization was performed prior to Principal Component Analysis (PCA) to ensure data comparability. The values of each indicator were uniformly transformed to the [0, 1] interval to achieve unit consistency. The specific normalization formula used is as follows:

12 $$X_{norm} = \frac{{X - X_{\min } }}{{X_{\max } - X_{\min } }}$$

where ${\mathrm{X}}_{{{\mathrm{norm}}}}$ represents the normalized index value, ${\mathrm{X}}$ represents the original index value, and ${\mathrm{X}}_{{{\mathrm{max}}}}$ and ${\mathrm{X}}_{{{\mathrm{min}}}}$ represent the maximum and minimum values respectively within the study area.

(4) Principal component analysis

Following normalization, PCA consolidated the six indices into the first principal component (PC1), capturing the dominant variance pattern. PC1 weights were determined by both the contribution of each indicator to the principal component and its inherent characteristics, minimizing subjectivity in weight assignment^39,40^. PC1 weights were objectively determined by variable loadings. To establish positive ecological interpretability (higher values indicating better conditions), the calculated PC1 can be subtracted from 1 to obtain the initial ecological index IRSEI0.

13 $$IRSEI_{0} = 1 - \{ PC1\left[ {NDVI, WET, LST, NDBSI, CSI, SWCFI} \right]\}$$

To facilitate the measurement and comparison of the indicators, the IRSEI0 can also be normalized:

14 $$IRSEI = \frac{{IRSEI_{0} - IRSEI_{0\_\min } }}{{IRSEI_{0\_\max } - IRSEI_{0\_\min } }}$$

IRSEI is the established remote-sensing ecological index, and its value ranges from 0 to 1, with values positively correlating with ecological quality, where 1 and 0 represent optimal and degraded ecological states, respectively. Based on an established classification system, IRSEI values were categorized into five ecological grades: Poor (0, 0.2], Fair (0.2, 0.4], Moderate (0.4, 0.6], Good (0.6, 0.8], and Excellent (0.8, 1].

### Spatial autocorrelation analysis

Contemporary studies^41,42^ indicate that traditional remote sensing ecological index evaluations neglect spatial dimensions, whereas spatial autocorrelation analysis addresses this limitation. Spatial autocorrelation quantifies the degree of association between attribute values at a given geographical unit and those at neighboring units^43^. Quantifying IRSEI’s spatial dependence reveals ecological quality agglomeration patterns, spatial heterogeneity, and evolutionary characteristics that are otherwise obscured in traditional evaluations. Spatial autocorrelation analysis comprises global and local spatial autocorrelation analysis.

Global spatial autocorrelation assesses overall spatial interdependence across the study area. Moran’s I statistic quantifies global spatial association and the degree of spatial differentiation. Its calculation formula is as follows:

15 $$Global Moran{\prime} s I = \frac{{n\mathop \sum \nolimits_{i = 1}^{n} \mathop \sum \nolimits_{j = 1}^{n} w_{ij} (X_{i} - \overline{X})(X_{j} - \overline{X})}}{{\mathop \sum \nolimits_{i = 1}^{n} \mathop \sum \nolimits_{j = 1}^{n} w_{ij} \mathop \sum \nolimits_{i = 1}^{n} (X_{i} - \overline{X})^{2} }}$$

Local Indicators of Spatial Association (LISA) measure association strength between individual spatial units and their neighbors. LISA precisely identifies local clustering patterns and spatial heterogeneity^43^. The calculation formula for the local Moran’s I is as follows:

16 $$Local Moran{\prime} s I = \frac{{\mathop \sum \nolimits_{j = 1,j \ne i}^{n} w_{ij} (X_{i} - \overline{X})(X_{j} - \overline{X})}}{{\mathop \sum \nolimits_{i = 1}^{n} (X_{i} - \overline{X})^{2} }}$$

where, ${\mathrm{n}}$ is the number of spatial observations in the study area; ${\mathrm{X}}_{{\mathrm{i}}}$ and ${\mathrm{X}}_{{\mathrm{j}}}$ are the values of the ${\mathrm{ith}}$ and ${\mathrm{jth}}$ observations at the spatial location, respectively; ${\overline{\mathrm{X}}}$ is the average observed value of all the objects; and ${\mathrm{w}}_{{{\mathrm{ij}}}}$ is the spatial weight matrix, which represents the neighbourhood relationship of the ${\mathrm{ith}}$ and ${\mathrm{jth}}$ observations at the spatial location. The value range of Moran’s I is between -1 and 1. When objects ${\mathrm{i}}$ and ${\mathrm{j}}$ are adjacent, ${\mathrm{w}}_{{{\mathrm{ij}}}} { = 1}$; when objects ${\mathrm{i}}$ and ${\mathrm{j}}$ are non-adjacent, ${\mathrm{w}}_{{{\mathrm{ij}}}} { = 0}$. If ${\text{I > 0}}$, it indicates positive spatial correlation, suggesting that the research objects are clustered in space. The larger the value, the more pronounced the positive spatial correlation. When ${\text{I < 0}}$, it indicates negative spatial correlation, meaning that the research objects are dispersed in space. The smaller the value, the greater the spatial disparity. If ${\text{I = 0}}$, it means that the research objects are randomly distributed and show no correlation.

To systematically assess spatial correlation in the SDYRB’s eco-environmental quality, spatial autocorrelation analysis was performed using IRSEI data from 2009, 2014, 2019, and 2023. Global autocorrelation was quantified using Moran’s I to assess significant spatial clustering of IRSEI values during each time period.

Then, to maintain consistency with the resampled multi-source driving factor data and avoid scale bias, while balancing the need to represent spatial heterogeneity with computational efficiency for a large watershed and the ability to identify local structures—and in accordance with general practices for spatial analysis of ecological environments at meso- and macro-regional scales^44,45^, this study employed a 1000 m × 1000 m grid sampling approach. Local spatial autocorrelation analysis employing Local Indicators of Spatial Association (LISA) identified hot spots (high-value clusters) and cold spots (low-value clusters), enabling detailed assessment of spatial aggregation patterns.

###  OLS

OLS is a widely used parameter estimation method for linear regression models^46^. In this study, a linear regression model was employed to examine both the magnitude and direction of the influence exerted by independent variables on the dependent variable. The method operates by minimizing the sum of squared residuals between the observed values and the predicted values, thereby determining the optimal regression coefficients. This approach allows for the quantification of the association between each predictor (driving factor) and eco-environmental quality.

17 $$y_{i} = \beta_{0} + \mathop \sum \limits_{i = 1}^{k} \beta_{i} x_{i} + \varepsilon_{i}$$

In the formula, ${\upbeta }_{{0}}$ is a constant, ${\upbeta }_{{\mathrm{i}}}$ is the regression coefficient, and ${\upvarepsilon }_{{\mathrm{i}}}$ is the random error.

### GD

GD is a widely applied statistical method for detecting spatial differentiation patterns and their drivers. It shows high applicability in research areas including land use transformation, eco-environmental evolution, and cultivated land change^47^. This study employs two core modules—differentiation and factor detection, and interaction detection—to systematically examine the drivers of regional eco-environmental changes. Specifically, the q-value quantifies the explanatory power of a driving factor X for the spatial differentiation of attribute Y (the dependent variable), and its calculation formula is as follows.

18 $$q = 1 - \frac{1}{{N\sigma^{2} }}\mathop \sum \limits_{h = 1}^{L} N_{h} \sigma_{h}^{2}$$

In the formula, ${\mathrm{N}}_{{\mathrm{h}}}$ represents the number of samples at a specific level ${\mathrm{h}}$; ${\mathrm{N}}$ represents the total number of samples in the entire study area; ${\upsigma }_{{\mathrm{h}}}^{{2}}$ and ${\upsigma }^{{2}}$ represent the variances at the specific level ${\mathrm{h}}$ and in the entire study area ${\mathrm{Y}}$, respectively. The value of q ranges from 0 to 1. A larger q value indicates a higher degree of spatial differentiation of attribute Y. When the stratification is based on independent variable X, a larger q value indicates a stronger explanatory power of X for attribute Y^32^^.^

Interaction detection identifies the interactions between different driving factors X. Specifically, it evaluates whether the combined effect of factors X1 and X2 increases or decreases their explanatory power for dependent variable Y, or if their effects on Y are independent. The types of interaction relationships are shown in Table 4

**Table 4:** Interaction relationships among different factors.

Judgment basis	Interaction
$q(X1 \cap X2) < \min (q(X1),q(X2))$	Nonlinear weakening
$\min (q(X1), q(X2)) < q(X1 \cap X2) < \max (q(X1), q(X2))$	Single-factor nonlinear weakening
$q(X1 \cap X2) > \max (q(X1), q(X2))$	Two-factor enhancement
$q(X1 \cap X2) = q(X1) + q(X2)$	Independent
$q(X1 \cap X2) > q(X1) + q(X2)$	Nonlinear enhancement

## Results

### PCA of eco-environmental quality indicators

Using PCA, this study quantitatively derived the IRSEI for the SDYRB from 2009 to 2023. As shown in Table 5, the variance contribution rate of the first principal component (PC1) exceeded 70% in the study area.

**Table 5:** PCA of each index.

Year	Load values of each indicator on PC1						Eigenvalues	Contribution rate/%
	NDVI	WET	LST	NDBSI	CSI	SWCFI	PC1	PC1
2009	0.427	0.123	− 0.366	− 0.253	− 0.325	0.441	0.0433	71.80
2014	0.460	0.184	− 0.429	− 0.271	− 0.374	0.478	0.0431	73.74
2019	0.461	0.137	− 0.391	− 0.221	− 0.248	0.481	0.0425	77.71
2023	0.409	0.118	− 0.433	− 0.255	− 0.343	0.539	0.0434	71.07

Consequently, PC1 can effectively represent the majority of the characteristics captured by the six indicators. Analysis of the component loadings revealed that NDVI, WET, and SWCFI had positive ecological effects, whereas NDBSI, LST, and CSI had negative ecological effects, which is consistent with theoretical expectations. Therefore, the IRSEI model developed in this study is scientifically sound and applicable, providing a reliable reflection of eco-environmental quality in the SDYRB.

### Spatial distribution characteristics of the eco-environmental quality

**Fig. 3:**
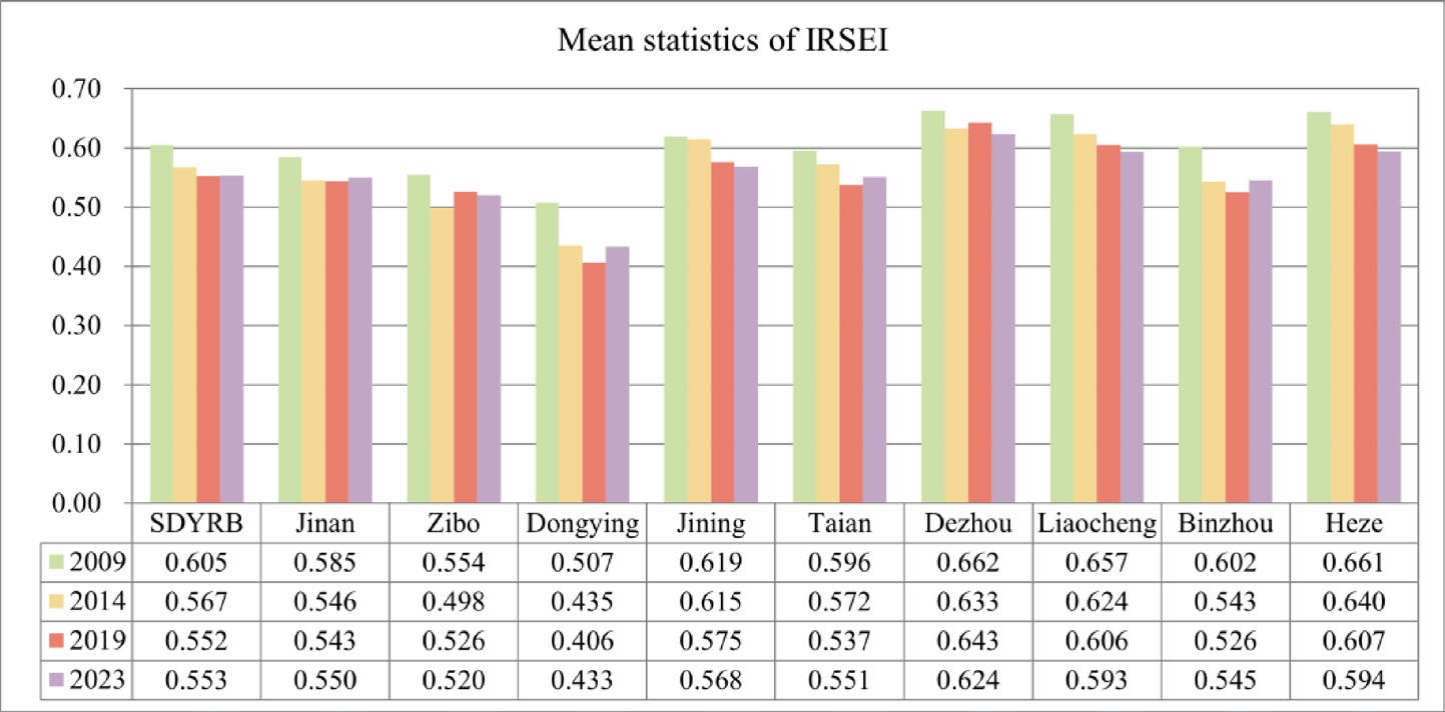
Mean RSEI values across different regions in SDYRB.

**Fig. 4:**
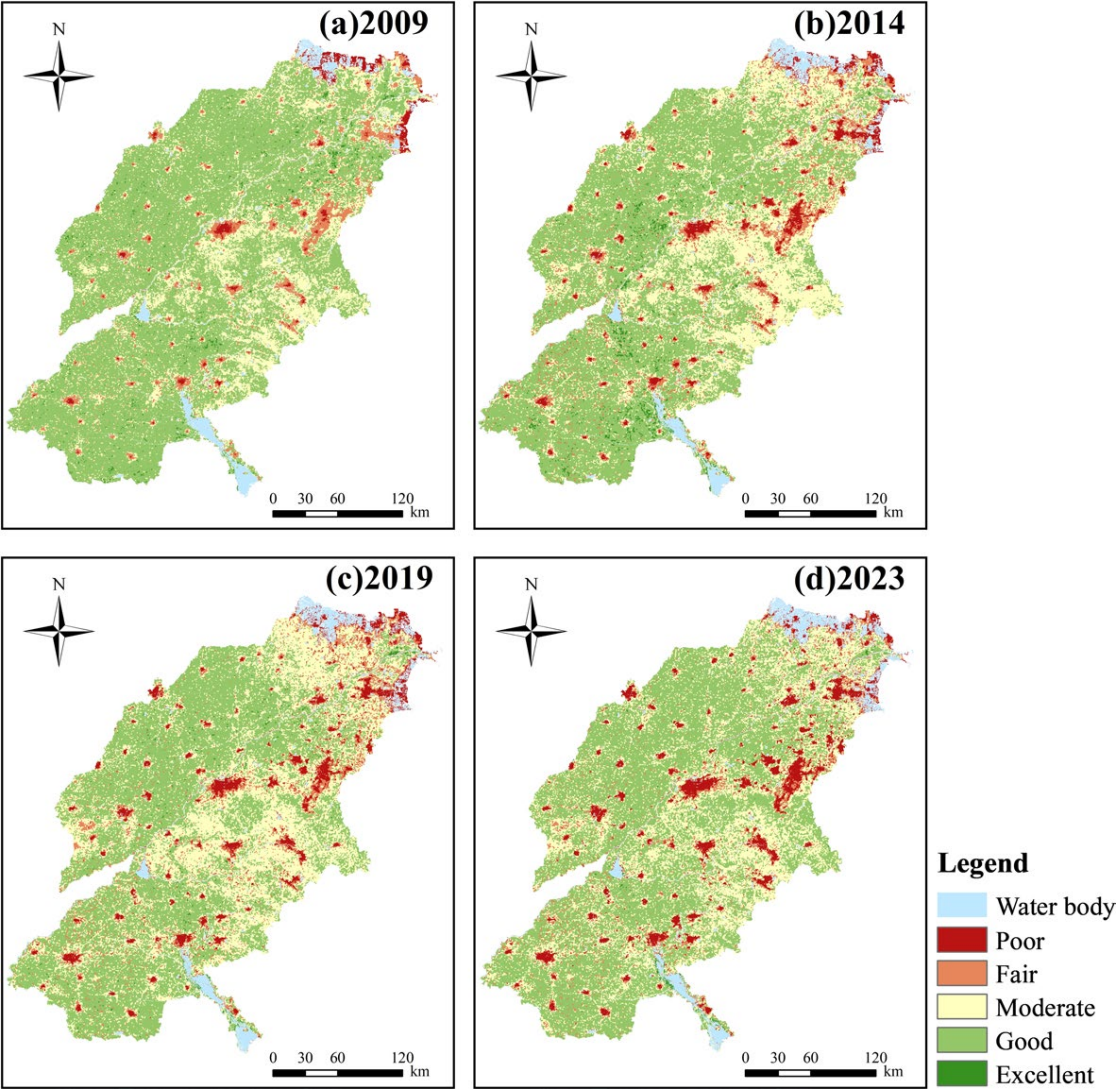
Spatial distribution of IRSEI in SDYRB.

**Fig. 5:**
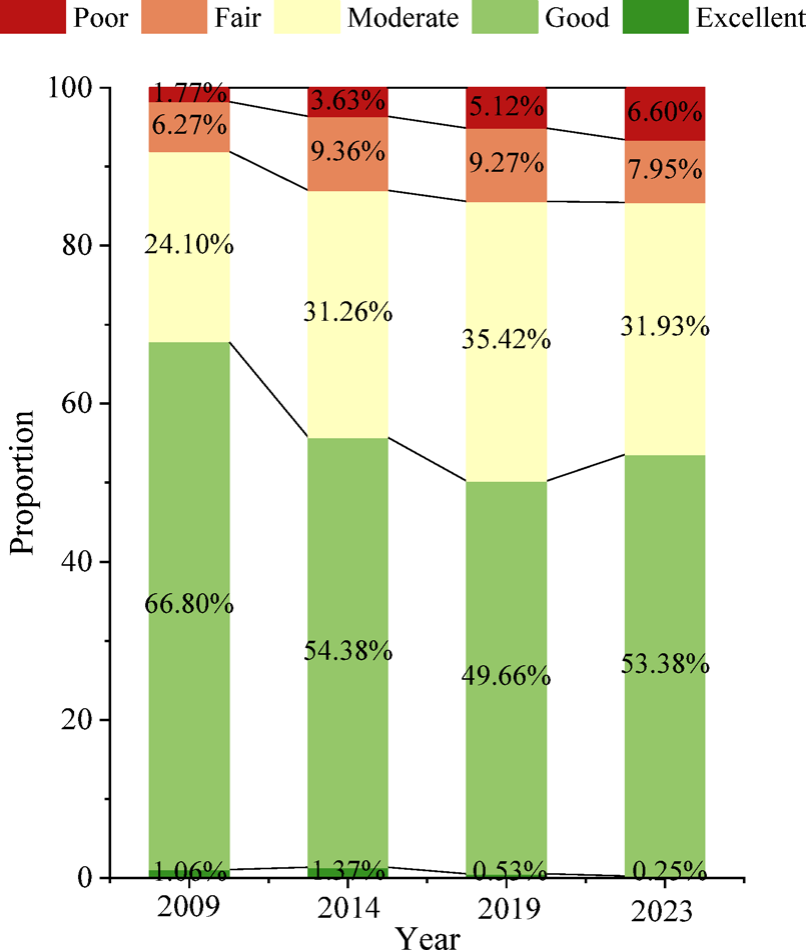
The proportion of IRSEI grades.

To facilitate a comparative analysis of the eco-environmental quality across different regions of SDYRB and over time, the mean IRSEI values for each region were calculated (Fig. 3). Subsequently, the annual IRSEI values were classified into five distinct levels: Poor [0, 0.2], Fair (0.2, 0.40], Moderate (0.40, 0.60], Good (0.60, 0.80], and Excellent (0.80, 1] (Fig. 4). The proportional area coverage of each level was then calculated for each year (Fig. 5). Between 2009 and 2023, the regional mean IRSEI fluctuated between 0.552 and 0.605, exhibiting an overall declining trend, with particularly notable degradation observed in the Yellow River Delta area. The mean value dropped from 0.605 in 2009 to 0.567 in 2014 and further to 0.552 in 2019, before experiencing a slight recovery to 0.553 in 2023. At the prefecture-level city scale, Jinan, Zibo, Dongying, Tai’an, and Binzhou exhibited a recovery pattern characterized by an initial decline followed by an increase. Among these, Dongying, Binzhou, and Tai’an showed significant rebounds. In contrast, Jining, Liaocheng, and Heze underwent continuous degradation, with Liaocheng experiencing the most substantial decline.

Spatially, the ecological quality generally exhibited a pattern of “superior in the west and poorer in the east” across the study years. In 2009, the overall ecological quality was relatively good, with a gradient of degradation from southwest to northeast, with poorer areas in the west appearing as scattered distributions (Fig. 4a). From 2009 to 2014, alongside rapid industrialization and urbanization in Shandong Province, ecological pressure sharply increased in economically developing areas such as Zibo, Jinan, and Tai’an. Large areas of the eastern region experienced a decline in ecological grade from Moderate to Poor, and areas of poorer quality evolved from scattered patterns into clustered distributions (Fig. 4b). Between 2014 and 2019, although environmental policies began to take effect, persistent extensive development patterns and non-point source pollution remained serious issues. Ecological degradation continued in the east, and most northern regions shifted from Good to Moderate (Fig. 4c). From 2019 to 2023, driven by the national strategy for ecological protection and high-quality development in the Yellow River Basin, industrial transformation and ecological restoration projects demonstrated preliminary results. Areas in central and northeastern parts improved from Moderate to Good, though some local areas continued to expand as Poor (Fig. 4d), indicating significant imbalance and complexity in regional ecological recovery. This spatial pattern of “superior in the west and poorer in the east” is closely linked to regional natural background conditions and socioeconomic patterns. The specific mechanisms will be analyzed in detail in the Discussion section.

To facilitate a more intuitive analysis of the inter-annual changes in IRSEI, the areas of eco-environmental quality grades were statistically analyzed for each year (Fig. 5). Specifically, from 2009 to 2014, areas classified as Poor, Fair, and Moderate increased significantly, while the area classified as Good decreased significantly, indicating a marked deterioration in eco-environmental quality. Between 2014 and 2019, areas of Poor and Fair grades remained relatively stable, whereas the area of Moderate grade increased, resulting in a slight overall decline in quality. From 2019 to 2023, the area classified as Good increased significantly, and the overall eco-environmental quality showed a tendency towards stabilization.

Overall, from 2009 to 2023, the Good grade consistently constituted the largest proportion, accounting for 49–66% of the study area. The proportions of the Poor and Excellent grades were relatively small. Notably, the proportion of the Poor grade, while being the smallest, exhibited a year-by-year increasing trend. The Fair grade proportion showed an initial sharp increase followed by a slight decrease. Conversely, the Excellent grade proportion displayed an initial slight increase followed by a sharp decrease. The Good grade proportion demonstrated a sharp initial decrease followed by a slight increase. The Moderate grade proportion exhibited an overall increasing trend. Collectively, these trends indicate a transition of areas from the Excellent and Good grades toward the Poor and Fair grades, signifying an overall decline in the eco-environmental quality of the SDYRB from 2009 to 2023.

### Spatial autocorrelation analysis of the eco-environmental quality

**Fig. 6:**
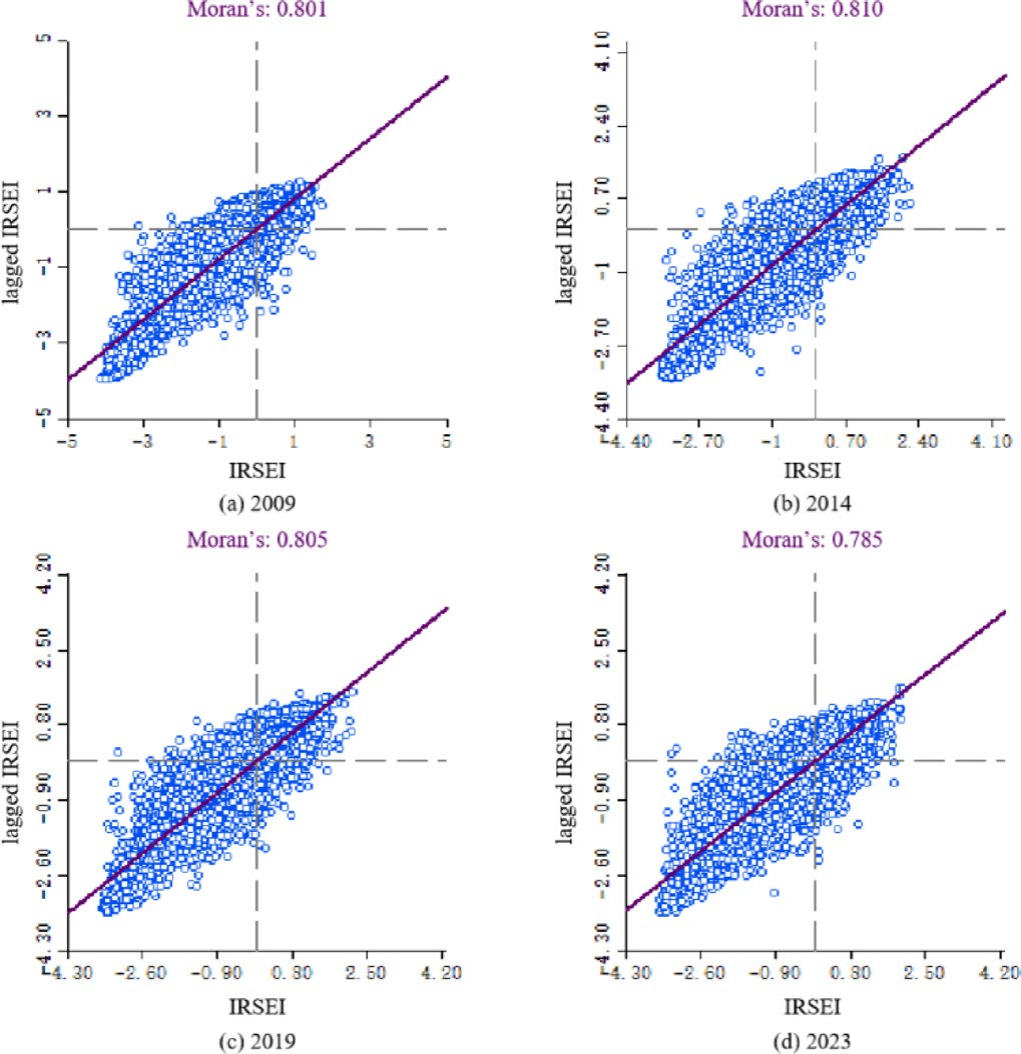
Moran’s I scatter plot.

To investigate the spatial distribution characteristics of eco-environmental quality within the SDYRB, spatial autocorrelation analysis of the IRSEI was conducted using Moran’s I scatter plots for the years 2009, 2014, 2019, and 2023. The results (Fig. 6) reveal that the annual Moran’s I indices ranged from 0.785 to 0.810, and all corresponding *P*-values were less than 0.05. This indicates a significant positive spatial autocorrelation of IRSEI within the study area, suggesting that regions with similar eco-environmental quality exhibit spatial clustering.

Specifically, in 2009, Moran’s I was 0.801, with scatter points concentrated predominantly in the first and third quadrants. This distribution reflects distinct clustering characteristics, where high-value areas are adjacent to other high-value areas and low-value areas are adjacent to other low-value areas. In 2014, Moran’s I increased to 0.810, and the point distribution exhibited tighter clustering along the regression line, indicating enhanced spatial aggregation. In 2019, Moran’s I was 0.805, decreasing to 0.785 in 2023. Despite these minor fluctuations, a strong positive spatial autocorrelation persisted throughout the study period. This indicates that the fundamental spatial clustering pattern of eco-environmental quality in the SDYRB remained relatively stable.

**Fig. 7:**
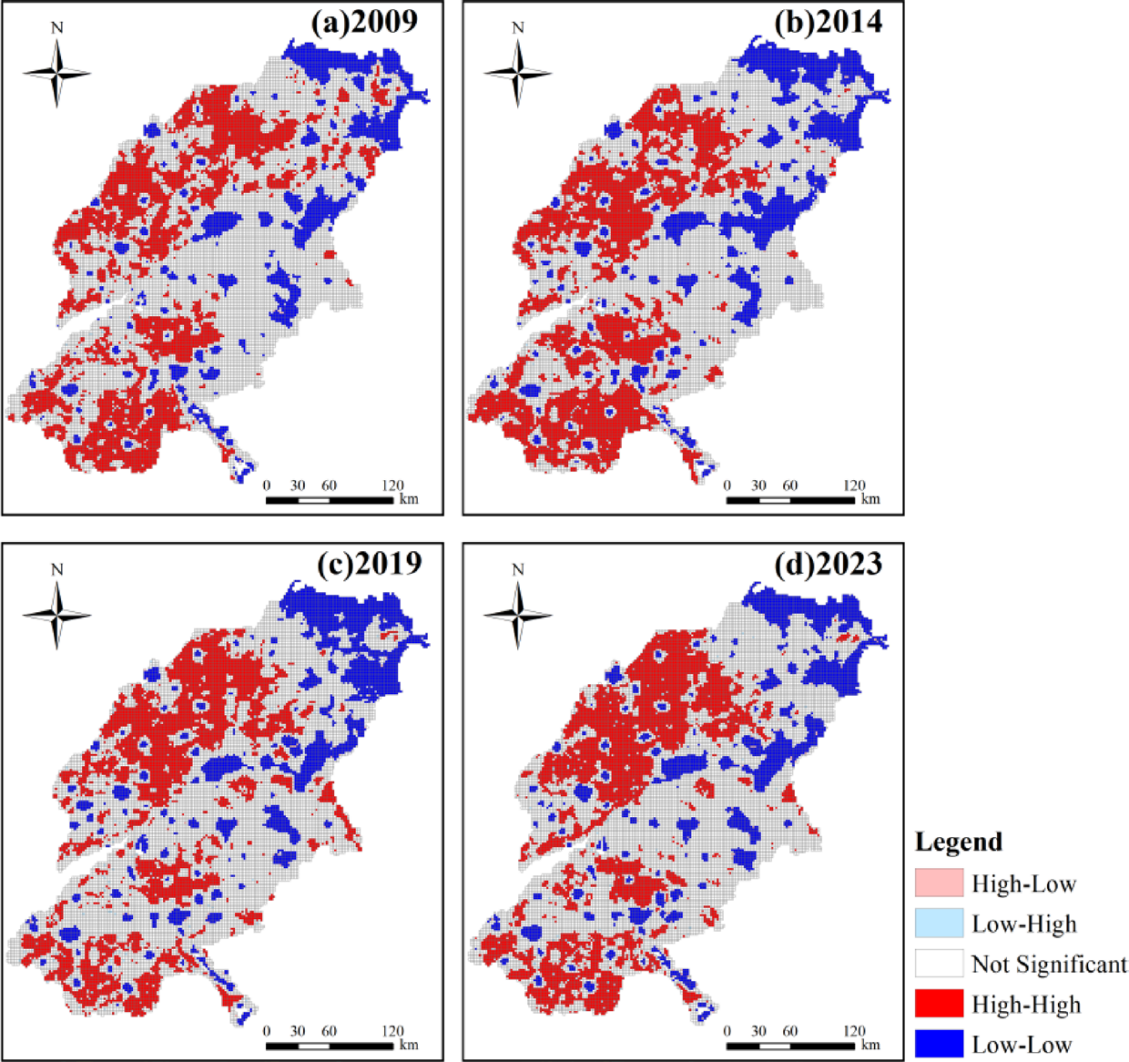
Spatial distribution of LISA index.

To further elucidate the spatiotemporal distribution characteristics of IRSEI in the SDYRB, this study employed local Moran’s I analysis to systematically examine the spatial clustering patterns (Fig. 7). The areal extent and proportional coverage of different cluster types were also calculated (Table 6). The analysis revealed that H–L and L–H clusters occupied relatively small and scattered areas. In contrast, H–H and L–L clusters covered larger, more contiguous areas. Across all study periods, the dominant spatial clustering patterns in the SDYRB were H–H and L–L clusters, underscoring the prevalence of positive spatial autocorrelation. The proportional coverage of both L–H and H–L types remained below 1%, indicating that negative spatial autocorrelation was not a prominent feature. This suggests a relative scarcity of spatial outliers attributable to the heterogeneity of regional IRSEI values.

**Table 6:** The area and proportion of different clustering features.

Year	2009		2014		2019		2023	
Class	Area/km^2^	Proportion/%	Area/km^2^	Proportion/%	Area/km^2^	Proportion/%	Area/km^2^	Proportion/%
Not significant	44,344.49	56.31	41,341.75	52.65	42,074.24	53.64	42,547.79	54.39
H–H	22,875.26	29.05	23,687.79	30.17	23,219.00	29.60	21,666.85	27.70
L–L	10,442.16	13.26	12,593.46	16.04	12,151.86	15.49	13,050.45	16.68
L–H	764.88	0.97	641.42	0.82	646.22	0.82	647.87	0.83
H–L	326.20	0.41	253.58	0.32	341.68	0.44	309.04	0.40

The H–H clusters were predominantly concentrated in the western and southern regions of the SDYRB. However, their areal extent decreased from 22,875.26 km^2^ in 2009 to 21,666.85 km^2^ in 2023, with the proportional coverage declining slightly from 29.05 to 27.70%. Although H–H clusters remained a significant positive spatial autocorrelation type, their spatial footprint contracted. This indicates that the agglomeration areas with high IRSEI values have become more scattered, potentially resulting from widening developmental disparities around some high-value areas, which may have weakened synergistic effects on regional eco-environmental improvement.

Conversely, L–L clusters were primarily located in northeastern Dongying City, central Jinan City, and central Zibo City. Over time, these low-value clusters exhibited a tendency to expand into surrounding areas. The area occupied by L–L clusters increased consistently, rising from 10,442.16 km^2^ in 2009 to 13,050.45 km^2^ in 2023. Consequently, their proportional coverage grew from 13.26 to 16.68%. This indicates a strengthening of clustering among areas with low IRSEI values. Such areas may be constrained by factors such as the natural environmental background and developmental path dependency, resulting in limited synergistic capacity for eco-environmental quality enhancement and a propensity to form contiguous low-value zones.

## Discussion

### Evolutionary process of the ecological environment in the SDYRB

**Fig. 8:**
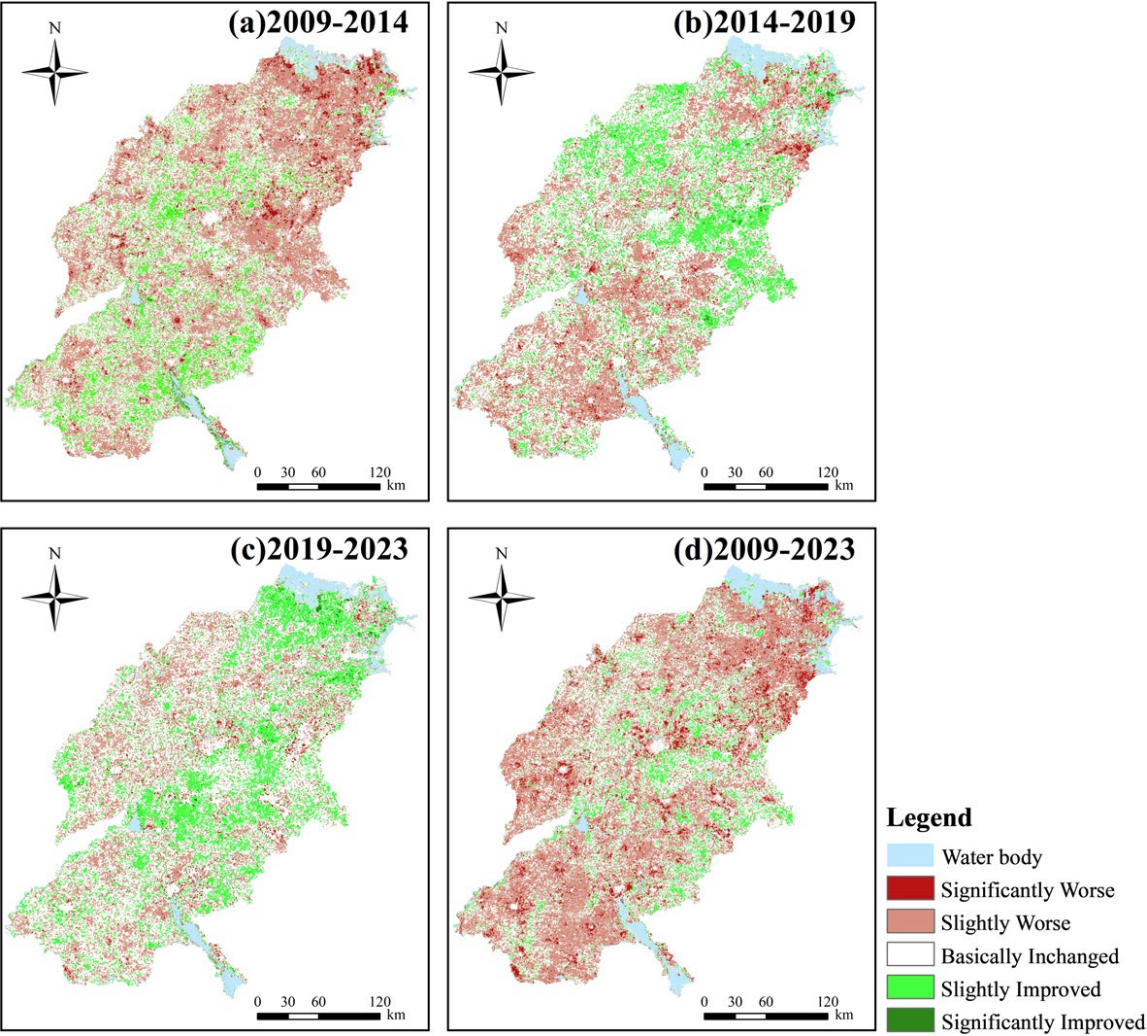
Spatial distribution of IRSEI change characteristics.

**Fig. 9:**
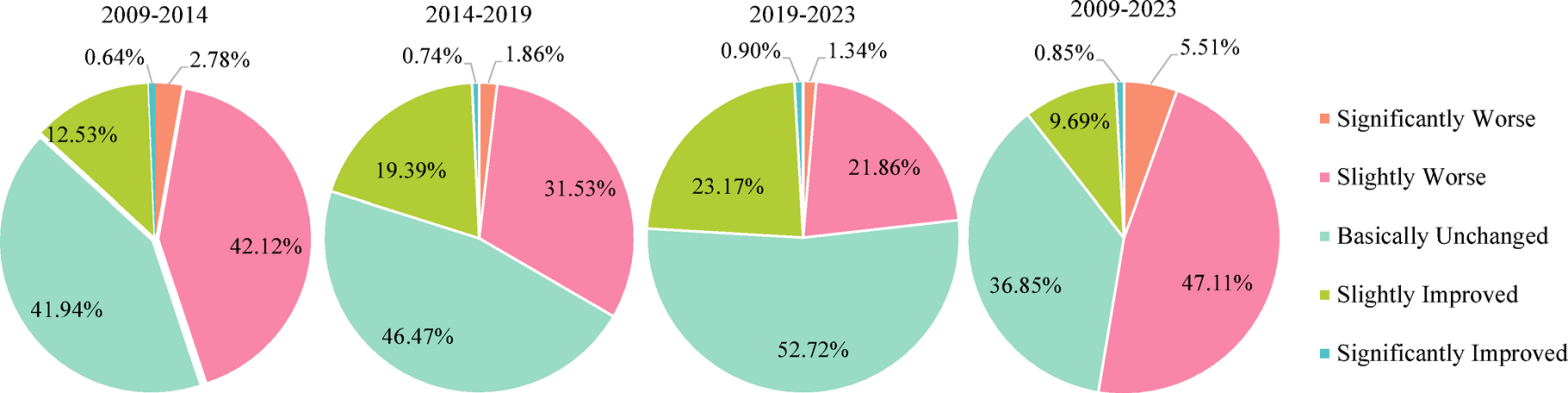
The proportion of IRSEI change amplitude.

Through temporal analysis and grade transfer calculations of the IRSEI at four time points (2009–2023), this study reveals a significant fluctuating trajectory of deterioration–mitigation–partial improvement in the ecological quality of the SDYRB (Figs. 8, 9). This evolutionary trajectory not only documents the region’s transition from an economy-first to an ecology-first development paradigm but also reflects the complex feedback mechanisms between natural environmental baseline conditions and human activities. The entire process exhibits remarkable spatial heterogeneity, with the overall pattern of “superior in the west and poorer in the east” representing a direct manifestation of the spatial coupling between fragile natural conditions and differentiated intensities of human disturbance. This evolutionary process can be deconstructed into three distinct characteristic stages.

**Fig. 10:**
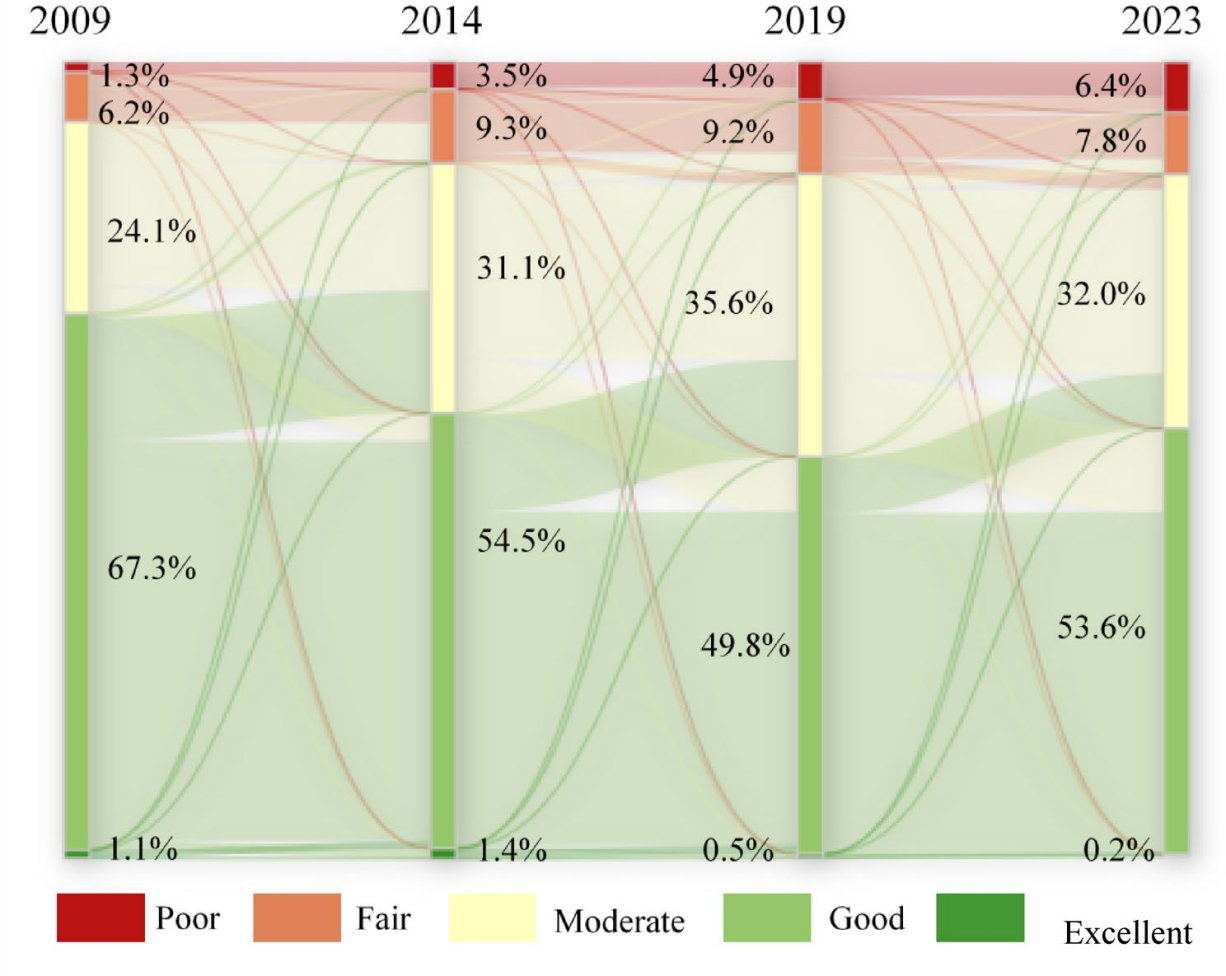
The Sankey diagram showing IRSEI grades of 2009–2023.

The period from 2009 to 2014 marked a phase of rapid deterioration, representing a concentrated manifestation of ecological overspend under the extensive GDP-oriented development model. During this stage, ecological environment quality exhibited a significant declining trend, with pixels classified as Slightly Worse and Basically Unchanged accounting for as much as 84.06% of the total (Fig. 9). More critically, the Sankey diagram (Fig. 10) reveal large-scale degradation of Excellent (91.7% transferred out) and Good (approximately 11,981 km^2^ downgraded to Moderate) ecosystems. This indicates that the ecosystem degradation was structural, and the loss of highly functional ecosystems posed a serious threat to regional ecological security. The core drivers of this sharp degradation stemmed from three interacting mechanisms. First, the landscape fragmentation mechanism driven by rapid industrialization and urbanization, where the drastic expansion of construction land physically encroached upon ecological spaces, severing ecosystem integrity and connectivity from a landscape ecology perspective, thereby undermining their critical functions^48^. Second, the compound pollution stress mechanism arising from the expansion of heavy industrial bases, epitomized by cities like Dongying and Binzhou. Here, industrial point-source pollution converged with non-point source risks from western agricultural areas, directly harming vegetation and disrupting soil ecology through the soil–plant system^49,50^. Finally, the water resource competition and imbalance mechanism was particularly pronounced in the vulnerable northern coastal areas. Human activities diverted water away from ecological uses, exacerbating groundwater over-extraction and soil salinization, which further diminished the resilience of the already fragile natural systems^51^.

The period from 2014 to 2019 marked a phase of decelerated ecological degradation, signaling an initial alleviation of ecological pressures under policy interventions. During this stage, the pace of ecological deterioration slowed markedly, with the proportion of pixels categorized as Basically Unchanged rising to 46.47%—constituting the dominant category—while the proportion classified as Slightly Improved increased to 19.39% (Fig. 9). This shift closely aligns with the implementation of stringent environmental policies around 2015, notably *the Water Pollution Prevention and Control Action* Plan, which imposed structural constraints on industrial point-source pollution through measures such as shutting down, suspending, merging, or relocating highly polluting enterprises^52^. However, the Sankey diagram (Fig. 10) indicate that the area of Good-grade ecosystems downgraded to Moderate-grade remained the largest, underscoring a pronounced lag effect in ecosystem recovery. Persistent historical pollution, widespread agricultural non-point source pollution, and the yet-to-be fundamentally transformed energy structure collectively maintained substantial overall ecological pressure, leaving the system in a fragile, sub-healthy equilibrium state.

The period from 2019 to 2023 witnessed a positive turning point in ecological quality, initiating a new phase of systematic restoration under national strategic guidance. During this period, Basically Unchanged (52.72%) and Slightly Improved (23.17%) categories became the absolute dominant trends, with the area of improvement exceeding that of degradation for the first time (Fig. 9). The Sankey diagram (Fig. 10) reveals an encouraging signal. Large-scale bidirectional conversions occurred between Good and Moderate grades, indicating the ecosystem transitioned from a unidirectional degradation trajectory to an active phase of dynamic recovery. This fundamental shift benefited from the top-down impetus of the major national strategy *Ecological Protection and High-quality Development in the Yellow River Basin*. Ecological governance in Shandong Province subsequently evolved from passive response to proactive systematic restoration. *The Integrated Protection and Restoration Project of Mountains, Rivers, Forests, Farmlands, Lakes, Grasslands, and Deserts* broke through the limitations of single-element management^53^. For instance, in the Yellow River Delta, ecological water replenishment practices helped regulate regional water-salt balance and alleviate soil salinization, demonstrating the effectiveness of multi-process coordinated governance. Simultaneously, the deepened implementation of *the New-old Kinetic Energy Conversion strategy* fundamentally reduced source pressures from resource consumption and pollution emissions at the developmental model level, establishing a solid foundation for sustained ecological improvement^54^.

This spatiotemporal evolutionary process ultimately shaped and reinforced the “superior in the west and poorer in the east” spatial differentiation pattern. In the western region (e.g., Heze and Liaocheng), the agricultural-dominated human activity pattern, while potentially contributing to non-point source pollution, has established extensive crop coverage that functions as a semi-natural anthropogenic vegetation system. This system provides crucial surrogate ecological functions in maintaining surface greenness and soil–water conservation^55,56^. Furthermore, its location within the Yellow River alluvial plain with superior natural endowments and relatively low industrialization pressure has enabled the persistence of these ecological advantages. In contrast, the eastern and northern coastal areas exhibit persistently low ecological quality with slow recovery, representing a typical case of resonance between inherently fragile natural conditions and high-intensity human disturbance. The region’s inherent soil salinization and poor vegetation site conditions constitute a fragile baseline characterized by low ecological thresholds. When superimposed with high-intensity disturbances such as heavy industrial development and urban built-up expansion, these conditions readily trigger virtually irreversible ecosystem degradation^57,58^. Although recent remediation measures have demonstrated efficacy, the notably slower recovery rate compared to other regions corroborates the significant spatial heterogeneity in ecosystem resilience.

In summary, the ecological evolution in the SDYBR over the past fifteen years serves as a vivid chronicle of human–environment interactions. Its trajectory of deterioration–mitigation–partial improvement clearly delineates the transitional pathway from growth-at-environmental-cost to the pursuit of a green development paradigm. Furthermore, the “superior-west-poorer-east” spatial differentiation pattern revealed in this study presents an evolutionary case distinct from the core-periphery concentric structures observed in the Yangtze River Delta^59^ and Pearl River Delta^60^. This demonstrates that in river basins characterized by high natural heterogeneity and diverse development models, ecological patterns emerge from the nonlinear coupling of multiple driving forces—including natural constraints, traditional agricultural practices, and intensive industrialization—rather than being solely attributable to urban expansion. This epistemological deepening necessitates that future ecological restoration and management must transcend universal governance templates. By fully accounting for ecosystem hysteresis, spatial heterogeneity, and the complex interactions among multiple drivers, we should implement more targeted, zonal governance strategies based on coupled natural-social system typologies^61^, thereby achieving sustainable regional human-nature development.

### Analysis of driving mechanisms for the ecological environment in the SDYRB

**Fig. 11:**
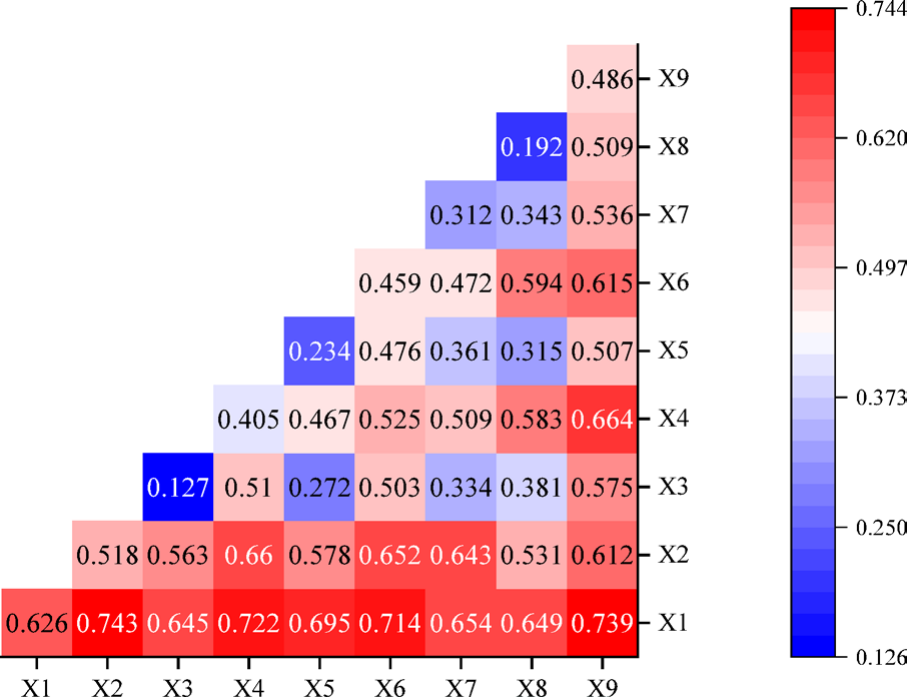
Interaction detection results of dual factors. Where X1, X2, X3, X4, X5, X6, X7, X8, and X9 denote mean annual temperature, mean annual evapotranspiration, annual precipitation, slope, soil moisture, land use, population density, scenic spot density, and nighttime light intensity respectively.

**Fig. 12:**
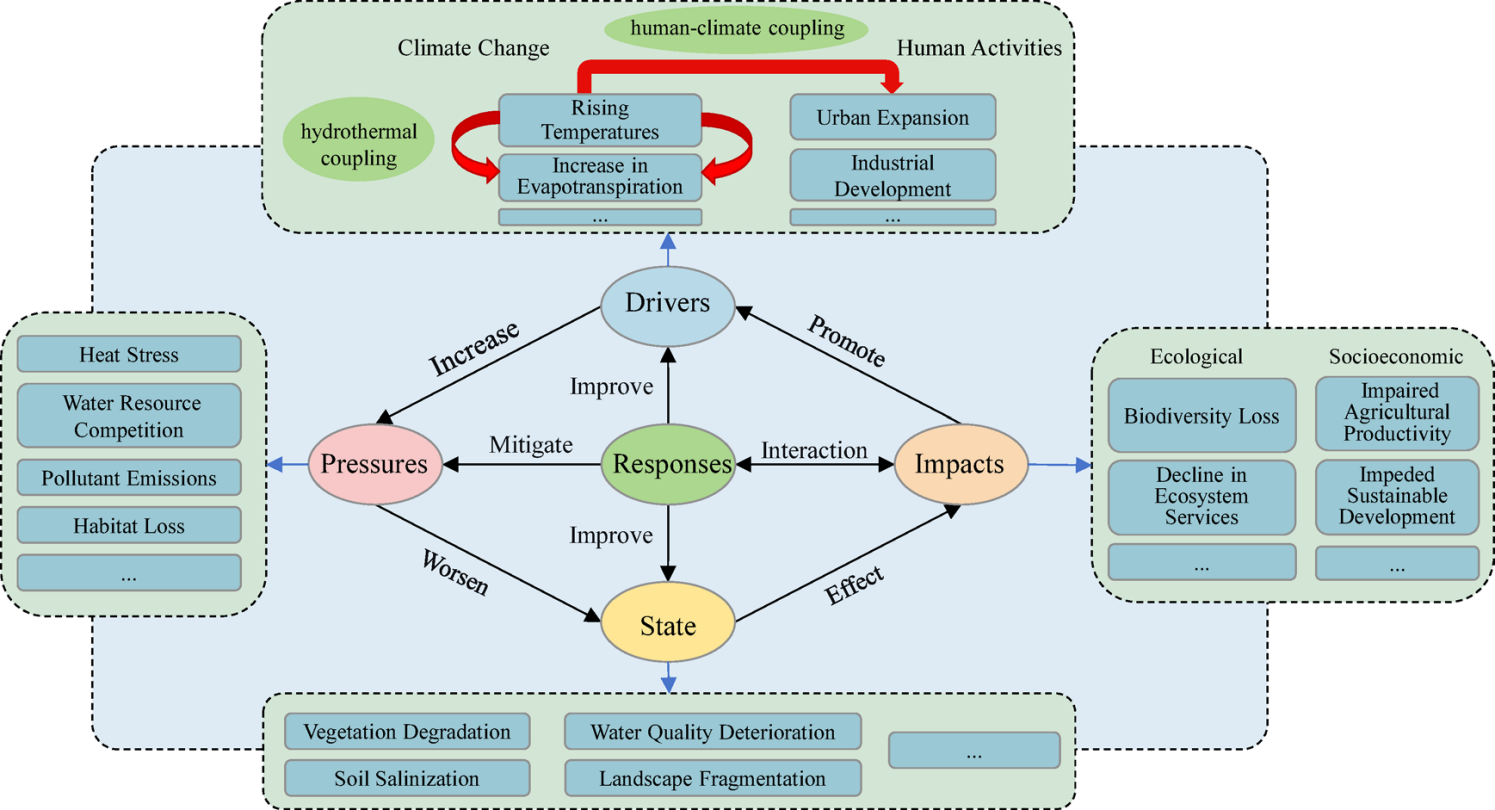
The framework for the evolution of the ecological environment in the SDYRB.

To thoroughly investigate the causes underlying the spatial heterogeneity of the ecological environment in the SDYRB, this study employed both OLS regression and the Geodetector model. The OLS model (R^2^ = 0.6224) and Geodetector results collectively identified mean annual temperature, mean annual evapotranspiration, and nighttime light intensity as the three core factors driving the spatial differentiation of IRSEI (Tables 7, 8). However, the pivotal finding of this study lies in the interaction detection results from the Geodetector (Fig. 11), which demonstrate that all factor interactions exhibit either two-factor enhancement or nonlinear enhancement, with explanatory power far exceeding that of any single factor. This clearly reveals that the ecological environment in the SDYRB constitutes a typical complex adaptive system. To synthesize these findings and systematically elucidate the causal relationships and feedback loops among the various elements, this study constructed a conceptual framework for the evolution of the ecological environment in the SDYRB (Fig. 12).

**Table 7:** OLS Estimation results of driving factors.

Variable	Coefficient	Standard deviation	*P*-value	VIF
Mean annual temperature	− 0.040643	0.000021	0.000000*	2.596413
Mean annual evapotranspiration	0.001285	0.000016	0.000000*	2.455101
Annual precipitation	0.000090	0.000002	0.000000*	1.707536
Slope	0.002168	0.031745	0.000000*	1.389358
Soil moisture	0.000045	0.000219	0.036984*	1.023979
Land use	0.009320	0.000033	0.000000*	1.190439
Population density	− 0.000041	0.002559	0.000000*	1.687626
Scenic spot density	− 0.003927	0.000333	0.000005*	1.371042
Nighttime light	− 0.008115	0.000671	0.000000*	1.837333

**Table 8:** Single factor detection results.

Parameter	Mean annual temperature	Mean annual evapotranspiration	Annual precipitation	Slope	Soil moisture	Land use	Population density	Scenic spot density	Nighttime light
q	0.626	0.518	0.127	0.405	0.234	0.459	0.312	0.192	0.486
p	0.000	0.000	0.000	0.000	0.000	0.000	0.000	0.000	0.000

Among these interaction mechanisms, several key coupling processes predominantly shape the regional ecological pattern. The interaction between mean annual temperature and evapotranspiration (hydrothermal coupling) demonstrates the strongest explanatory power among all factor combinations, highlighting the fundamental role of energy-water balance processes in configuring regional ecological patterns. In the SDYRB, particularly in the Yellow River Delta with severe groundwater over-extraction, intensified evapotranspiration under elevated temperatures directly aggravates soil salinization and vegetation drought stress, leading to a spiraling increase in ecological vulnerability. This finding aligns with related studies by Fu^5^ and Yao^22^ in arid and semi-arid regions, indicating that water stress serves as a critical bridge connecting climate change and ecological responses.

Furthermore, the interaction between mean annual temperature and nighttime light intensity (human-climate coupling) reveals a critical positive feedback loop. Under elevated temperature conditions, intensive energy consumption and anthropogenic heat emissions in urban and industrial areas (high nighttime light zones) significantly exacerbate the urban heat island effect^62^. This not only directly alters local microclimates and disrupts species phenology but also further increases cooling energy demand during summer, leading to additional heat and greenhouse gas emissions that collectively amplify the ecological negative impacts of regional climate change^63^. This mechanism underscores that the superimposed effects of climate warming and anthropogenic heat emissions constitute a crucial driver of ecological degradation in rapidly urbanizing regions^32^.

Additionally, the significant interaction between temperature and land use demonstrates how natural background conditions modulate the ecological effects of human activities. Under high-temperature conditions, agricultural expansion and increased impervious surfaces substantially alter surface albedo and energy partitioning, leading to enhanced sensible heat flux, aggravated local warming, and accelerated soil moisture depletion. Conversely, ecological land covers (e.g., woodlands, wetlands) can mitigate thermal stress through evaporative cooling effects^64^. Notably, this coupling effect exhibits significant spatial heterogeneity: in the ecologically vulnerable Yellow River Delta, such interactions may trigger vicious cycles of degradation, whereas in the relatively stable southern mountainous areas of Jinan, vegetation’s regulatory functions can partially buffer climatic pressures. This finding deepens our understanding of how climate background modulates the environmental effects of land use/cover changes.

These interacting mechanisms collectively form a core network driving regional ecological environment evolution. As illustrated in Fig. 12, the conceptual framework developed in this study integrates the Drivers-Pressures-State-Impacts-Responses (DPSIR) model with complex system theory^65^. It incorporates identified key drivers (e.g., climate change, human activities), their induced ecological pressures (e.g., thermal stress, water resource competition), resulting environmental state changes (e.g., vegetation degradation, soil salinization), consequent ecological and socio-economic impacts, and corresponding governance responses (e.g., environmental policies, ecological engineering) into a unified systemic framework. This schema particularly highlights two core mechanisms. Firstly, the hydrothermal coupling relationship between thermal stress and water resource competition. Secondly, the human-climate positive feedback loop formed between thermal stress and urbanization. The framework demonstrates that any change in regional ecological status results from multi-pathway, non-linear interactions, while human governance measures essentially represent a process of dynamic interplay with this complex natural-social system.

### Comprehensive implications and management significance

The analysis of driving factors in this study, particularly the revelation of the universal two-factor enhancement interaction, not only deepens the understanding of ecological issues in the SDYRB but also provides important empirical evidence and theoretical supplementation to the classic human-land system coupling theory^66^. Traditional human–environment relationship studies often focus on either natural subsystems (e.g., climate, hydrology) or human subsystems (e.g., land use, economic density), predominantly employing linear additive models to interpret their combined effects^67^. Our findings robustly demonstrate profound nonlinear synergistic effects between natural and anthropogenic elements. For instance, the interaction between mean annual temperature and nighttime light intensity reveals a typical positive feedback loop, indicating that under climate change scenarios, the ecological negative impacts of human activities are not merely additive but can be dramatically amplified. This recognition necessitates abandoning the reductionist view that treats human activities solely as external stressors, instead reconceptualizing them as endogenous variables deeply coupled with natural systems and mutually reinforced through feedback mechanisms. Consequently, the findings of this study provide significant implications for regional ecological governance, spanning from theoretical understanding to practical implementation.

In terms of governance paradigms, this study strongly advocates for a fundamental shift from single-element management toward systemic governance. Traditional reactive and symptom-focused approaches, by neglecting the interactive enhancement effects among driving factors, often yield localized and limited outcomes that may even be counteracted by negative effects from other coupled processes. Future efforts must transition to systemic governance that addresses the interactions among multidimensional factors such as climate-human-topography. Specifically, management strategies should be more targeted and tailored according to the dominant interaction types in different regions. For instance, in human-climate coupling hotspots (e.g., urbanized high-temperature zones like Jinan and Zibo), priority should be given to planning urban ventilation corridors and increasing blue-green infrastructure (e.g., parks, wetlands) to mitigate the urban heat island effect and break the positive feedback loop between energy consumption and heat emissions. Conversely, in agricultural areas and ecologically fragile zones dominated by water-thermal coupling (e.g., the Yellow River Delta), comprehensive measures such as water-saving irrigation, conservation tillage, and ecological water replenishment should be prioritized to enhance the fundamental climate resilience of ecosystems.

At the theoretical level, this study not only provides empirical validation for the human-land system coupling theory but also makes contributions to its advancement. Traditional research on human–environment relationships has predominantly focused on linear superposition or unidirectional influences between subsystems. However, our findings, by revealing the universal pattern of two-factor enhancement, provide compelling evidence for profound nonlinear synergistic effects between natural and anthropogenic subsystems. This implies that neglecting these interactive effects may lead to substantial underestimation of the lever effect of human activities under specific natural backgrounds (e.g., anthropogenic heat emissions in high-temperature zones), or the constraining effect of natural limitations on human interventions (e.g., the impediment of fragile basal conditions to ecological restoration). This recognition necessitates that future research must transcend simple correlation analysis and embrace complex system methodologies capable of characterizing feedback loops.

In conclusion, identifying dominant factors and their interaction mechanisms is a prerequisite for implementing precise, efficient, and sustainable watershed ecosystem management. The ecological conservation and high-quality development of the SDYRB must be grounded in respecting natural laws, particularly the principles governing the interactions of multiple stress factors. Through systematic territorial spatial planning and ecological restoration projects that holistically coordinate natural and anthropogenic elements, a fundamental transformation can be achieved—shifting from reactive response to proactive planning, and from localized improvement to systematic enhancement.

## Conclusions

This study addressed the prominent issues of salinization and soil erosion in the SDYRB by developing an Improved Remote Sensing Ecological Index (IRSEI) that integrates the Composite Salinity Index (CSI) and Soil–Water Conservation Function Index (SWCFI). This enabled a systematic assessment of the spatiotemporal evolution patterns and driving mechanisms of regional ecological environment quality from 2009 to 2023. The main conclusions and contributions are as follow.

1. The regional ecological environment quality underwent a fluctuating trajectory of deterioration-mitigation-partial improvement during the study period, exhibiting a stable spatial heterogeneity pattern characterized as “superior in the west and poorer in the east”. This pattern results from the resonance between the fragile natural baseline in the east and high-intensity human disturbance, combined with the relatively superior agricultural ecological foundation in the west.
2. Two-factor enhancement or nonlinear enhancement effects were prevalent observed between natural and anthropogenic factors. Specifically, the hydrothermal coupling of temperature ∩ evapotranspiration and the human-climate positive feedback loop of temperature ∩ nighttime light emerged as core mechanisms driving ecological patterns and amplifying negative environmental impacts. These findings clearly demonstrate that climate warming significantly intensifies the ecological stress caused by human activities, while the effectiveness of ecological restoration is profoundly modulated by the resilience of the natural baseline.
3. Ecological governance in the SDYRB requires a transition from single-element management to systemic governance, with particular focus on factor interactions. For instance, in human-climate coupling hotspots, efforts should prioritize planning ecological corridors and expanding blue-green infrastructure to disrupt the urban heat island positive feedback cycle. Conversely, in ecologically vulnerable areas under water-thermal stress, implementing water-saving irrigation and ecological water replenishment should be prioritized. Future watershed management must respect and leverage these complex interaction patterns. Only through systematic optimization of territorial spatial planning and ecological engineering can we achieve a fundamental transformation from localized improvement to systemic enhancement, thereby supporting ecological conservation and high-quality development in the SDYRB.

## Data Availability

The authors confirm that the data supporting the findings of this study are available within the article.
